# A novel chemogenomics analysis of G protein-coupled receptors (GPCRs) and their ligands: a potential strategy for receptor de-orphanization

**DOI:** 10.1186/1471-2105-11-316

**Published:** 2010-06-10

**Authors:** Eelke van der Horst, Julio E Peironcely, Adriaan P IJzerman, Margot W Beukers, Jonathan R Lane, Herman WT van Vlijmen, Michael TM Emmerich, Yasushi Okuno, Andreas Bender

**Affiliations:** 1 Division of Medicinal Chemistry, Leiden/Amsterdam Center for Drug Research, Leiden University, Einsteinweg 55, 2333CC, The Netherlands; 2 Leiden Institute for Advanced Computer Science, University of Leiden, The Netherlands; 3 Department of PharmacoInformatics, Center for Integrative Education of Pharmacy Frontier, Graduate School of Pharmaceutical Sciences, Kyoto University, Kyoto, Japan; 4 Unilever Centre for Molecular Science Informatics, Department of Chemistry, University of Cambridge, Cambridge, UK

## Abstract

**Background:**

G protein-coupled receptors (GPCRs) represent a family of well-characterized drug targets with significant therapeutic value. Phylogenetic classifications may help to understand the characteristics of individual GPCRs and their subtypes. Previous phylogenetic classifications were all based on the sequences of receptors, adding only minor information about the ligand binding properties of the receptors. In this work, we compare a sequence-based classification of receptors to a ligand-based classification of the same group of receptors, and evaluate the potential to use sequence relatedness as a predictor for ligand interactions thus aiding the quest for ligands of orphan receptors.

**Results:**

We present a classification of GPCRs that is purely based on their ligands, complementing sequence-based phylogenetic classifications of these receptors. Targets were hierarchically classified into phylogenetic trees, for both sequence space and ligand (substructure) space. The overall organization of the sequence-based tree and substructure-based tree was similar; in particular, the adenosine receptors cluster together as well as most peptide receptor subtypes (*e.g*. opioid, somatostatin) and adrenoceptor subtypes. In ligand space, the prostanoid and cannabinoid receptors are more distant from the other targets, whereas the tachykinin receptors, the oxytocin receptor, and serotonin receptors are closer to the other targets, which is indicative for ligand promiscuity. In 93% of the receptors studied, de-orphanization of a simulated orphan receptor using the ligands of related receptors performed better than random (AUC > 0.5) and for 35% of receptors de-orphanization performance was good (AUC > 0.7).

**Conclusions:**

We constructed a phylogenetic classification of GPCRs that is solely based on the ligands of these receptors. The similarities and differences with traditional sequence-based classifications were investigated: our ligand-based classification uncovers relationships among GPCRs that are not apparent from the sequence-based classification. This will shed light on potential cross-reactivity of GPCR ligands and will aid the design of new ligands with the desired activity profiles. In addition, we linked the ligand-based classification with a ligand-focused sequence-based classification described in literature and proved the potential of this method for de-orphanization of GPCRs.

## Background

G protein-coupled receptors (GPCRs) comprise a large family, more than 800 in human [1], of cell surface receptors that consist of seven transmembrane (TM) helices. These receptors are activated by a variety of external stimuli, including light, ions, small molecules, lipids, and proteins; moreover, the majority of therapeutic drugs act on GPCRs [2]. Because of the limited number of target crystal structures [3-6], GPCR drug design relies largely on ligand-based approaches [7] such as property-based methods [8], pharmacophore models [9], and substructure methods [10]. These methods do not require any knowledge about the target protein; however, combining them with target information often increases their potential. The resulting so-called 'chemogenomics' approaches thus involve both ligand-based and target-based aspects [11]. They do not focus on a single group of ligands and one individual target, but rather on groups of ligands against groups of targets. The central idea is that similar targets have similar ligands [12,13]. Therefore, relationships between targets from the sequence side can be exploited to search for novel receptor ligands on the chemical structure side.

Traditionally, the GPCR superfamily has been classified based on sequence homology of the receptors. Kolakowski grouped all seven transmembrane (7-TM) proteins into classes A to F for receptors proven to bind G-proteins and class O for the other 7-TM proteins [14]. Class A receptors resemble rhodopsin and form the largest cluster. Later, Fredriksson *et al. *proposed a more elaborate classification for known and predicted human GPCRs [1]. Surgand *et al. *presented a sequence-based phylogenetic classification of GPCRs viewed from a ligand perspective [15]. By selecting residues pointing inwards into the generic binding pocket of GPCRs, the authors assembled a set of 30 residues most likely to be accessible for ligand binding. Based on these residues, phylogenetic clustering was performed. Although only a subset of residues was used, the classification was similar to classifications based on the full sequence. Applications of a grouping such as proposed by Surgand *et al. *constitute ligand design for related receptors, as well as de-orphanization of GPCRs [15]. However, the study by Surgant *et al. *is somewhat limited by the scarcity of structural protein data where the identification of binding site residues was solely based on the structure of bovine rhodopsin. It could not yet take into account recent advances that yielded three pharmacologically relevant X-ray crystal structures, namely those of the human β_2 _and turkey β_1 _adrenoceptors, as well as of the human adenosine A_2A _receptor [3,5,6,16]. Building further on Surgand's work, Gloriam *et al. *proposed an extended set of ligand-accessible residues, derived from visual inspection of the newly available X-ray GPCR crystal structures, from supporting mutagenesis data and from the evaluation of previously established residue sets [17]. The resulting set of 44 residues was then applied to cluster class A GPCRs into a phylogenetic tree, which reflected similarities in binding site of the receptors.

Complementary to these sequence-based classifications are the ligand-based classifications of GPCRs. Approaches that use ligand similarity measures for target classification have been previously described [18,19]. Keiser *et al. *related targets by pair-wise comparison of their ligands [20]. From a set of 65 k ligands, a network was constructed connecting almost all 246 targets through sequential linkage. From this, previously unknown antagonism of methadone on the muscarinic M_3 _receptor and of emetine on the α_2_-adrenoceptor was identified.

While sequence-based similarity relies on comparison of the residues at certain positions in the sequence, there is no unambiguously defined method to measure ligand-based similarity. One way of defining ligand similarity is to consider the overlap of substructures in the molecules. Frequent substructure mining is a method for finding the most common substructures in a set of molecules [21-23]. It evaluates all possible substructures, not only discrete fragments that are present in the molecules; it is therefore an exhaustive approach, resulting in a more complete view on the structural features in the set.

In this study, we employ frequent substructure mining to determine the similarity between groups of ligands in a thorough and unbiased manner. This substructural similarity is then used for classification of GPCRs according to relatedness of substructure profiles of their ligands. The substructure-based classification of GPCRs visualizes relatedness of receptors in the form of a phylogenetic tree, which is then compared to the sequence-based phylogenetic classifications of GPCRs. The differences in tree organization are examined with methods that visualize changes in target position. Taken together, we present a (GPCR) classification from the small molecule (ligand) perspective, which facilitates analysis of target similarities and differences in ligand-binding behavior. In addition, we explore the potential of our ligand-based classification in receptor de-orphanization, *i.e*. the prediction of new ligands for orphan receptors.

## Results and Discussion

### Sequence-based classification

**Figure 1:**
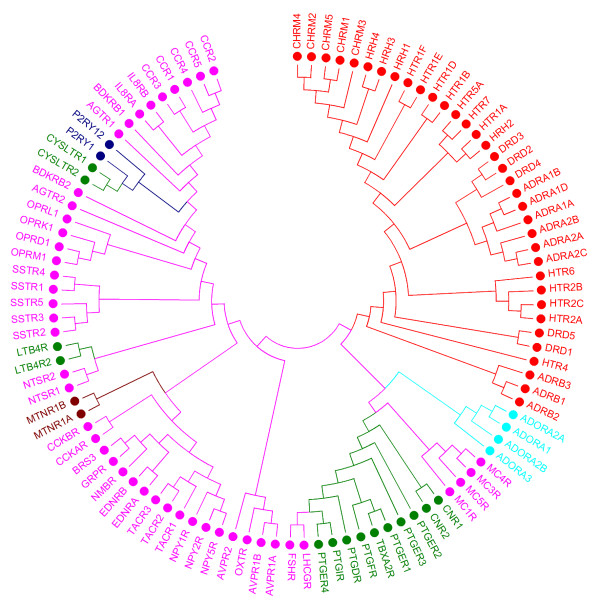
**Phylogenetic tree of human Class A GPCRs based on sequence information (44 residues of the GSK set)**. Human Class A GPCRs are clustered based on the 44 ligand-binding residues as defined in the GSK set. Subfamilies are color-coded according to ligand type whereby the broad ligand types applied by Gloriam *et al. *[17] were used. red - receptor with aminergic ligands; pink - peptide ligands; green - lipid ligands; dark blue - purinergic P2Y ligands; light blue - adenosine ligands; brown - melatonin ligands.

Three types of sequence-based phylogenetic trees were built, namely: one tree that was based on the full 7-TM sequence, one tree employing 30 residues described by Surgand *et al. *[15], and one tree which was based on the set of 44 residues described by Gloriam *et al. *[17]. Note that the three sequence-based trees presented here are different from those published in the referenced original work [1,15,17], since in the current study orphan receptors, receptors with a low number of ligands, and singleton receptors were left out. Singleton receptors are receptors that are the only (available) member in their respective subfamily. Due to the chemogenomic nature of this study, we focus on the phylogenetic tree based on the set of Gloriam *et al. *since it represents the ligand perspective best; this set is referenced as the GSK set [17]. The two other trees are provided for reference purposes in Additional file 1 - Phylogenetic trees based on 7TM domain and selected residues. The tree that was built based on the multiple sequence alignment of the GSK set is shown in Figure 1. The GPCR subtypes in this tree are grouped as branches in the tree according to subfamily and target since it resembles the sequence-based phylogenetic tree on which GPCR classification is based [1]. For instance, the opioid receptor subtypes δ, κ, μ, and NOP cluster together, as well as the α- and β-adrenoceptor subtypes. The fact that clustering follows the receptor classification is expected since the classification of GPCRs was based on sequence similarity [24,25]. Four clusters are clearly defined in the tree: the aminergic receptors, the adenosine receptors, the prostanoid receptors, and the peptide-binding receptors.

### Ligand-based classification

**Figure 2:**
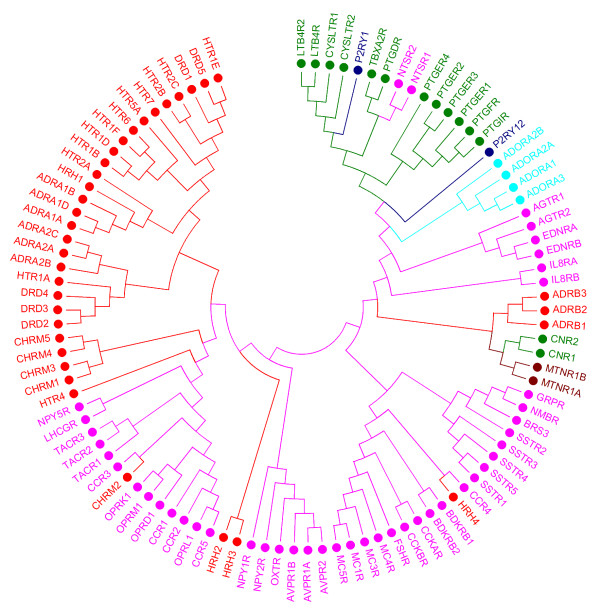
**Phylogenetic tree of human Class A GPCRs based on ligand information (frequent substructure mining)**. Human Class A GPCRs are clustered based on the frequent substructure analysis. Subfamilies are color-coded according to ligand type whereby the broad ligand types applied by Gloriam *et al. *[17] were used. red - receptor with aminergic ligands; pink - peptide ligands; green - lipid ligands; dark blue - purinergic P2Y ligands; light blue - adenosine ligands; brown - melatonin ligands.

The ligand-based receptor classification, which we will compare to the sequence-based classification, is provided in Figure 2. Subfamilies in this tree are more scattered; however, most subfamilies cluster together. For instance, except for the two purinergic receptors (P2Y_1 _and P2Y_12_) and the two glycoprotein hormone receptors (FSH and LH), all other receptors represented by only two subtypes, such as the melatonin or the leukotriene B_4 _receptors, are clustered together. The adenosine receptors A_1 _(ADORA1), A_2A _(ADORA2A), A_2B _(ADORA2B), and A_3 _(ADORA3) group together, indicating overlap in ligand profiles. This may imply that ligands for these receptor subtypes are non-selective, such as the adenosine receptor antagonists caffeine and theophylline. Additionally, receptor selectivity may vary with relatively small changes in ligand structure: an 8-cycloalkyl substituent on theophylline confers A_1 _receptor selectivity, whereas a phenylstyryl substituent on the same position in caffeine renders these compounds selective for the A_2A _receptor. The purinergic receptor P2Y_12 _is found near the adenosine receptors owing to the purine core typical for ligands of both these subfamilies. In agreement with the ligand selectivity reported for the α_1_-, α_2_-, and β-adrenoceptor subfamilies, these receptors form three distinct clusters [26]; furthermore, the α_1B _and α_1D _receptors are the closest in the distance matrix. The muscarinic acetylcholine receptors M_1_, M_3_, M_4_, and M_5 _(CHRM1/3/4/5, in Figure 2) cluster together as one group, supporting the low subtype selectivity of muscarinic antagonists [27]. However, the acetylcholine receptor M_2 _is found more distant from this cluster. This indicates the presence of distinct chemical classes in the ligand set of the M_2 _receptor, which may be the result of inclusion of allosteric ligands. For instance, gallamine is an allosteric modulator of the muscarinic M_2 _receptor [28] that is also present in the GLIDA database [29], classified as an M_2 _antagonist. In general, the remaining aminergic receptors (serotonergic, dopaminergic, histaminergic and cholinergic) are more scattered throughout the substructure tree. This means that targets share ligands or ligand substructures among subfamilies/subtypes, which is in line with the high level of polypharmacology observed for these aminergic GPCRs [30]. For instance, the serotonin receptor 5-HT_1A _clusters together with the D_2 _dopamine receptor, which fits with reports on antipsychotic compounds combining dopamine D_2 _receptor antagonism and serotonin 5-HT_1A _receptor agonism [31,32]. Structurally similar ligands may act on diverse targets, for instance, when ligands have a GPCR-privileged structure at their core [33,34]. The grouping of the eight prostanoid receptors (Figure 2) indicates similarity in substructure profiles of the ligands. This is based on the fact that most prostanoid receptor ligands are direct derivatives of the endogenous ligands [35,36], the so-called eicosanoids. These ligands are highly similar, all consisting of large aliphatic, lipophilic alkyl chains. The presence of the leukotriene and cannabinoid receptors in this lipid cluster may seem strange at first. Leukotrienes are however also eicosanoids, which clarifies the position of the leukotriene B_4 _and cysteinyl-leukotriene receptors in this cluster [37,38]. In addition, arachidonic acid is the common precursor for eicosanoids and two derivatives of arachidonic acid, anandamide and 2-arachidonylglycerol, both of which are endogenous ligands ('endocannabinoids') of the cannabinoid receptors.

The relationship between target clustering in the substructure tree (Figure 2) and ligand promiscuity suggests that the substructure tree may be used to identify possible side effects on receptors that are close neighbors in this tree. For instance, off-target activity of ligands can be identified. If inspection reveals a ligand to bind to receptor(s) that are phylogenetically related to the target of interest, a more detailed experimental follow-up with respect to receptor selectivity would be worthwhile.

### Tree comparison

Visual comparison of the sequence tree (Figure 1) with the substructure tree (Figure 2) reveals that the overall phylogenetic organization is similar. For instance, with the exclusion of the glycoprotein, P2Y, angiotensin, and bradykinin receptors, all other receptors represented by two subtypes occur in pairs in both the ligand tree and the sequence tree. This is also true for receptors with three subtypes present in the dataset, *e.g*. the three members of the α_1_, the α_2_, and the β_1 _adrenoceptors, as well as the bombesin receptors. Exceptions to this rule are the neuropeptide Y and vasopressin receptors. In addition, the prostanoid receptors largely group together in both trees, as do most of the aminergic receptors.

The clear distinction between the two dopamine receptor types, i.e. D_1 _and D_5 _(D_1_-like) versus D_2_, D_3_, and D_4 _(D_2_-like), exists both in the sequence-based classification and ligand-based classification. This is in agreement with a previous study [39] and also known from drugs on the market such as the benzazepines that favor D_1_-like over D_2_-like dopamine receptors. Similarly, antipsychotics such as chlorpromazine have a higher affinity for the D_2_-like subtypes than D_1_-like receptors [40].

**Figure 3:**
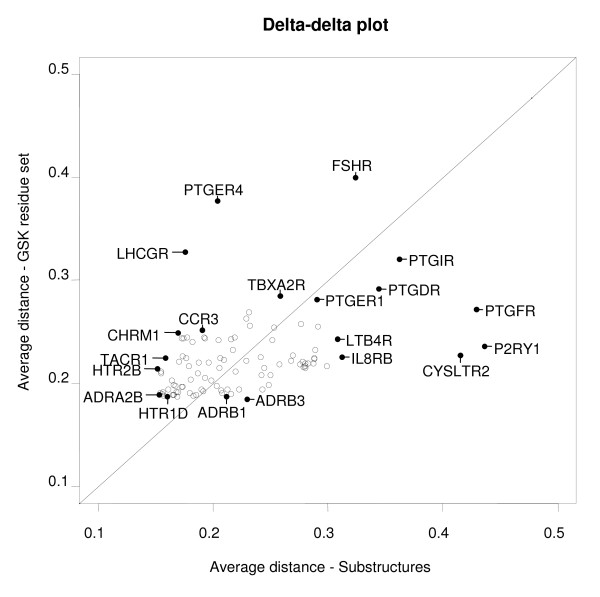
**Delta-delta plot visualization of receptor distances in sequence and substructure space**. The delta-delta plot visualizes how target distances differ between sequence-based classification (GSK set, y-axis) and substructure-based classification (x-axis). The average distance towards the other targets is plotted for sequence and substructure space. A few targets are highlighted in the plot to serve as examples. These are marked by a black dot and a label that denotes the gene symbol. Targets that are, on average, more distant from the rest are plotted further away from the origin; targets plotted above the diagonal are more distant in sequence space, while targets plotted below the diagonal are more distant in substructure space. For example, the FSH receptor (FSHR) is positioned relatively far from the origin and above the diagonal. This indicates that this receptor is, in general, more distant from the other receptors, most prominent in sequence space.

The fact that many clusters arise in both trees indicates that the receptors in these clusters have similar sequences and similar ligands, that is, ligands with substantially overlapping substructure sets. However, there are also receptor targets for which this is clearly not the case. The (qualitative) similarities and differences among sequence and substructure trees are discussed in the following. A delta-delta plot was constructed to compare how pairs of receptors change. This plot, provided in Figure 3 (and described in detail in the Materials and Methods section), visualizes how receptor distances deviate between the sequence-based tree and the ligand-based classification of receptors. In sequence space, receptor distances indicate the (dis)similarly between protein sequences, while in ligand space, receptor distances reflect the overlap in structural features found in ligands for these receptors. For each receptor, the mean distance to all other receptors is plotted. From the delta-delta plot, it becomes apparent that the prostanoid receptors and P2Y_1 _receptor are on average the most distant receptors from the rest of the classes. The distances of the purine P2Y_1 _receptor, the prostanoid FP receptor, and leukotriene receptor CysLT_2 _towards the other classes are all larger in substructure space than in sequence space, implicating that overall their ligands show little resemblance with ligands of the other GPCRs. In contrast, for most aminergic receptors, *e.g*. for the α_2B_-adrenoceptors and the 5-HT_2B _serotonin receptor in Figure 3, distances are smaller in substructure space compared to sequence space. This, again, corresponds with the high polypharmacology found for aminergic ligands, such as for most atypical antipsychotics [41], with clozapine as a prominent example [42]. With the exception of a few targets (FSH, LH), the distribution of targets in the delta-delta plot is more scattered along the x-axis (substructure space) than the y-axis (sequence space). This may be a reflection of the evolutionary relationship between sequences, which results in coverage of a small region of the overall sequence space. The ligands for these targets do not have such a direct relationship and thus cover a broader range in overall substructure space.

The difference between ligand-based and target-based classifications may be due to convergent evolution [43]. Functional convergence denotes how proteins that differ in sequence may fulfill the same protein function. The protein sequence of GPCR subtypes will be similar in parts that are involved in the endogenous ligand recognition but may be different in other parts, for instance those parts that play a role in recognition of other, exogenous, ligands (*e.g*. synthetic drugs). These may therefore have a different selectivity profile compared to the endogenous ligand.

### Validation

**Figure 4:**
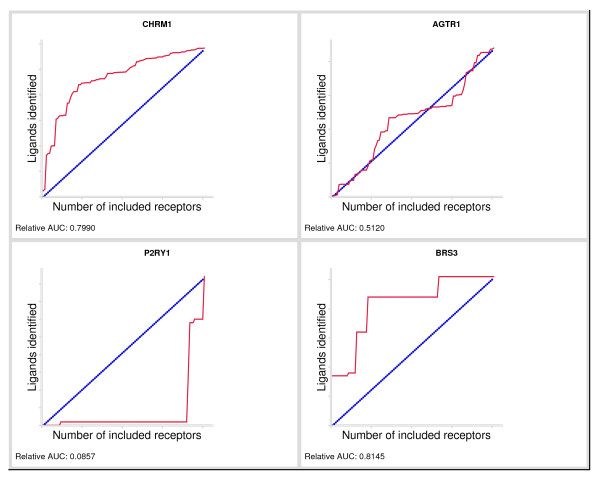
**Examples of plotted scores for the *leave-one-out *validation**. Example plots expressing the performance of the simulated receptor de-orphanization. Performance plots for the following receptors are provided (from left to right and from top to bottom): CHRM1 - muscarinic acetylcholine receptor M_1 _(first category); AGTR1 - angiotensin receptor AT_1 _(second category); P2RY1 - P2Y_1 _purinoceptor (third category); BRS3 - bombesin receptor BB_3 _(fourth category). These examples are discussed in the text. The full set of plotted scores is provided in Additional file 2 - Plotted scores for the *leave-one-out *validation. For each plot, receptors are ordered along the x-axis (labeled "Number of included receptors") in order of increasing distance in sequence space to the receptor under study. On the y-axis (labeled "Ligands identified"), the cumulative number of retrieved ligands is depicted, normalized linearly to the interval [0;1]. The red curve indicates the number of active ligands that are retrieved when including all (closest) receptors that are listed along the x-axis up to that point. For example, the plot of the muscarinic acetylcholine receptor M_1 _(CHRM1) displays a steeply rising curve near the origin, indicating that many of its ligands are retrieved using a small number of closest receptors. The blue diagonal illustrates recovery of ligands when performance is equal to random prediction. The relative area under the curve (AUC) of the red curve is stated at the bottom of each plot. An AUC above 0.5 indicates good performance, while poor performance is indicated by an AUC of 0.5 or below.

To validate how well our method performed as a chemogenomics method, *i.e*. how well it connects sequence space with small molecule space and how applicable the relationship is in practice, we conducted a 'virtual de-orphanization exercise'. For each receptor in the dataset, we pretended not to know any of its ligands by excluding them from the datasets (we 'orphanized' the receptor in this particular run of the protocol). We next predicted its ligands by considering a model derived from the closest neighbors of the receptor in sequence space (we attempted to 'de-orphanize' the receptor whose ligands we omitted from the study in the previous step). For this calculation, the distance matrix for the GSK residue set was used. The cumulative number of correctly identified ligands of every receptor is plotted against the number of closest neighbors (sequences) included to find these ligands. The (relative) area under the curve (AUC) and shape of the curve are measures of the performance of our method. In 93% of the studied receptors, de-orphanization of the pretended orphan receptor using the ligands of related receptors performed better than random (AUC > 0.5) and for 35% of receptors de-orphanization performance was good (AUC > 0.7). All AUC plots could be divided into four categories according to curve shape and AUC (the complete set of plotted scores is available as additional material in Additional file 2 - Plotted scores for the *leave-one-out *validation). Typical examples of the four categories are given in Figure 4. The first category is most abundant and consists of curves with a convex shape and an AUC above 0.5, marking good performance. An example of this category is the muscarinic acetylcholine receptor M_1 _(CHRM1 in Figure 4) with an AUC of 0.7990. Curves of the second category display a gradual rise that is approximately equal to the diagonal of the plot. These plots have an AUC near 0.5, indicating performance that is equal to random prediction. An example is the plot of the angiotensin receptor AT_1 _(AGTR1 in Figure 4) with an AUC of 0.5120. Curves of the third category perform worse than random and are characterized by a concave shape and an AUC below 0.5. Clearly the worst example is the P2Y_1 _purinoceptor with an AUC value of 0.0857 (P2RY1 in Figure 4). In contrast to the first three categories, curves of the fourth category do not have a clear AUC range. This category consists of curves that are divided into several discrete parts of alternating rises and plateaus, as shown in the plot of bombesin receptor BB_3 _(BRS3 in Figure 4), with an AUC of 0.8145. Performance varies from good (BRS3) to worse than random, depending on the value of the AUC. An example of such a plot with an AUC value below 0.5 is the FSH receptor (not shown, see: Additional file 2 - Plotted scores for the *leave-one-out *validation) with an AUC of 0.4428. The steep rises are caused by a few receptors identifying the majority of ligands. Some of these curves are steeply rising at the start, which suggests that part of its ligand set could be readily identified even though this is not reflected in the AUC. The poor performance concerning the P2Y_1 _receptor is probably due to the nature of its ligands: this set consists of a small number of highly similar ligands that all possess a phosphate group, a feature not found in other ligands in the database. The number of features (substructures) shared with ligands of this receptor and other receptors is therefore small. Interestingly, the adenosine A_1 _and A_3 _receptors, which are also purinergic, identify most (28 out of 42) of the P2Y_1 _ligands. However, in sequence space these receptors are at great distance (at positions 91 and 92, respectively).

Overall, our method proves useful for receptor de-orphanization, since for 93% of receptors studied de-ophanization performed better than random selection (AUC > 0.5) and for 35% of receptors de-orphanization performed well (AUC > 0.7).

### Limitations of the work

In the present study, some targets were excluded due to insufficient availability of ligand data in the source databases. The absence of a receptor may influence the order of other receptors in the trees. Scarcity of ligand data is reflected in the substructure profiles, thereby influencing the correlations among receptors. The issue of data (in) completeness and its effect on interaction networks was recently discussed by Mestres *et al. *[44]. Using three datasets of increasing complexity (more connections) that linked ligands to targets based on full chemical identity, the authors showed that an increase in the number of connections rapidly leads to shifts in connection patterns. However, our study linked targets based on overlap in substructures; as a consequence sharing of substructures rather than of ligands is sufficient for targets to be identified as related. Bender *et al. *and Keiser *et al. *already showed that overlapping ligands are not necessary to predict whether targets are close in ligand space [19,20]. In addition, our method employs an exhaustive approach to analyze the structural features of ligands. Frequent substructure mining considers all possible substructures that occur in the ligands and is therefore unbiased, *i.e*. all possible substructures were evaluated, not only those intuitive to chemists, such as functional groups, ring systems (e.g. a phenyl ring), and linkers [45]. However, in the present study less 'obvious' substructures such as ethyl or isobutyl are also considered [21]. For a complete discussion on substructure generation and evaluation, see ref. [46]. Our method is not limited to GPCRs alone; it is easily extended to other protein families for analysis of the differences between subfamily phylogenies, given that sufficient ligand information is available. For instance, it can be applied to the realm of enzymes to complement other chemogenomics analyses [47].

## Conclusions

In this work, we presented a ligand-based phylogenetic classification that complements the well-established sequence-based classification of proteins, and applied our method to classification of GPCRs. This alternate view may contribute to our understanding of GPCR classification since it reveals relationships that are unnoticed with conventional phylogeny. Targets were analyzed based on the substructure profiles of their ligands using an unbiased approach. The overall organization of the sequence tree and the substructure tree was similar; however, substantial differences were also discovered. In the substructure tree, several clusters of subtypes were identified. For instance, it was found that the adenosine receptors group together, and that certain GPCR subfamilies that do not share sequence homology cluster because of ligand similarity. Thus, receptor similarities that signal for potential off-target effects, such as for the serotonergic receptors, are readily identified. In addition, combined with sequence-based classification, the ligand-based classification presented has proven potential (93% of receptors with AUC > 0.5 and 35% with AUC > 0.7) for de-orphanization of receptors.

## Methods

### Datasets

#### Ligands

Ligands for human GPCRs were collected from three publicly available data sources: the StARLITe database, as made available by ChEBI (EMBL-EBI) as part of the ChEMBL database [48], GLIDA [29], and KiDB [49]. ChEMBL consists of a collection of more than 500,000 small molecules annotated with activity. Here, only activity values measured directly from binding studies were included. Compounds with K_i_, IC_50_, or EC values below 10 μM were considered active. GLIDA provides biological information on GPCRs (sequences) and chemical information about ligand structures. It has links to several external databases, GPCRDB [25], UniProt [50], PubChem [51], and DrugBank [52]. A reported affinity in one of these source databases classifies a compound as active, independent of the reported binding affinity. Ligands are annotated with an activity type, namely: full agonist, partial agonist, agonist, antagonist or inverse agonist. In the present study, we focused only on binding affinity and not on the activity type. This allowed us to merge the set with the rest of the data. KiDB provides information on drugs and molecular compounds that interact with GPCRs, ion channels, transporters, and enzymes. The entries in KiDB are annotated with ligand, K_i _value, radiolabeled ligand, receptor name, source & tissue, species, and PubMed link to the publication(s). Our dataset consisted of ligands from all three sources, by selecting human GPCR ligands with a molecular weight between 50 and 700 Da. Only targets that had 20 or more ligands listed were used. In this study, we focused on class A (rhodopsin-like) GPCRs since the majority of targets are from class A and only a minor part from class C; combining both classes would have negatively affected homogeneity of the phylogenetic trees, thereby hampering comparison. For the same reason, we removed two singleton targets (targets that are the only member in a subfamily), the gonadotrophin-releasing hormone receptor and the ghrelin receptor. The final set consisted of 102 targets (provided in Table 1 of Additional file 3 - List of GPCRs used in this study) with 37350 unique ligands in total.

#### Sequences

The multiple sequence alignment of (specific residues of) the 7-TM domain was obtained from GPCRDB [25,53]. Only human receptors that were non-olfactory and not orphan were used.

### Tree generation

#### Frequent Substructure Mining

For the ligands of each receptor, the most frequently occurring substructures were determined. This was accomplished by using the frequent subgraph-mining algorithm [54], which finds all frequent substructures in a set of molecular graphs [23]. For a description and a quantitative comparison of recent substructure mining algorithms, see [55]. Briefly, starting from the smallest substructure, namely the single atoms, the algorithm finds the number of molecules in which the substructure occurs. If this occurrence is above a user-defined minimum, the minimum support value, the substructure is stored. Stored substructures are stepwise extended, and tested in a systematic manner, with the aim of testing all possible substructures that have at least one of the stored substructures as their basis. The algorithm seeks ways to test only those substructures that actually occur in the set, and that have a frequency above the set minimum. An important concept of frequent substructure mining is the *a priori *principle, originating from frequent item set mining [56]. Algorithms based on the *a priori *principle exploit that the frequency of a substructure will be equal or lower than the frequency of the substructures it contains. Therefore, whenever the occurrence of a substructure is below the minimum support, all extensions of that substructure are discarded.

Structures were represented as labeled graphs with a special type for aromatic bonds. In this study, the minimum support value was set to 30% of the number of ligands in each activity set. At this value, the algorithm provided a large group of substructures while still being computationally feasible to work with. In addition, molecular structures were sorted in ascending order according to the number of bonds. This allowed the algorithm to prune scarce, complicated substructures that consisted of a large number of bonds, thereby reducing memory requirements. If the set of generated substructures is disproportionately large (more than 1000 times larger) compared to the majority of the other classes, the generated substructures are discarded except for those that also occur in other classes. This step was performed in order to prevent single targets from dominating the analysis. Since in practice most classes generated sets of less than 1000 substructures, a cut-off of 1 M substructures was used. Substructures with molecular weight below 50 Dalton were discarded. The frequent substructures of all classes were merged into one set, removing any duplicates. For all substructures in this set, the frequency in each subfamily was determined. To calculate the correlation between two targets, we used the substructure frequencies as features for that target. A correlation matrix was constructed by calculating the Pearson correlation coefficient for each pair of targets. Finally, a distance matrix was constructed by subtracting the values of the correlation matrix from unity and normalizing the results linearly to the interval [0;1].

#### Phylogenetic Trees

To study receptor organization, receptors were clustered into a phylogenetic tree using the Neighbor-Joining (NJ) method (Neighbor from the PHYLIP package [57]). This method infers phylogenies from the pair-wise distances between receptors. Phylogenetic trees built from distance matrices facilitate tree comparison across domains. In addition, NJ clusters each domain equally well since it does not involve an 'evolutionary clock', a concept rooted in evolutionary biology. Two distance matrices represented the similarities of the receptors: according to the frequent substructures of their ligands and the 7-TM domain sequence alignment, both were visualized as a phylogenetic tree, with receptors as leaves of the tree. The number of branches between two leaves in the tree grows with dissimilarity of these two leaves.

The protein distances between the aligned sequences were calculated with Protdist from the PHYLIP package version 3.6. using the Jones-Taylor-Thornton matrix (default) [57]. Both the sequence-based and ligand-based phylogenetic trees were constructed using the neighbor.exe program from the PHYLIP package. Tree construction might be influenced by the order in which targets are provided to the tree constructor. To minimize the influence on the resulting phylogenetic tree, target input order was randomized 10 times and 10 new trees were generated. From these, a consensus tree was built. MEGA4 [58] was used for editing the layout of the trees and for visualization. Trees were rooted on the mid-points, that is, a root is placed at the mid-point of the longest distance between two taxa of the unrooted tree. Taxa were arranged for balanced shape and trees were visualized as circular trees showing only topology, *i.e*. branch lengths do not reflect evolutionary distance in a quantitative manner.

### Tree comparison

For the comparison of trees, several methods and visualizations are available; however, there is not a single definitive measure for tree difference. To visualize how the receptor positions change between two trees we employed a delta-delta plot.

#### Delta-Delta plots

The delta-delta plot reveals how receptor locations behave globally with respect to the median of all receptors. It was used to visualize the differences in location of each receptor in sequence space and in substructure space. This plot is an adaptation from the delta-delta plot in Garr *et al. *[59]. It is a new way of tree comparison, which visualizes the differences among trees graphically, as opposed to the sole calculation of a numerical distance between two trees which is not trivial to interpret. For each receptor, the mean distance of that receptor to all other receptors was calculated. This value was plotted in a scatter plot, with each axis representing the mean distance of the respective node in one of the trees. The interpretation of this plot is as follows. Along both axes, receptors plotted far from the origin are, on average, more distant from the rest of the group, while receptors plotted close to the origin were closer to the rest of receptors. Receptors plotted near the diagonal do not change much in their mean distance to other receptors when going from one tree to the other (since they are close to the X = Y diagonal). Receptors plotted above or below the diagonal have different average distance to the other receptors between trees. For instance, consider a delta-delta plot that plots a substructure tree along the x-axis and a sequence tree along the y-axis. If a receptor is plotted above the diagonal, the mean distance of that receptor to the other receptors is larger in the sequence tree than the substructure tree; for receptors plotted below the diagonal, the opposite is true.

### Validation

#### Leave-one-out validation

This experiment is repeated for every receptor (the 'orphan receptor') by temporarily removing ligands of this receptor from the dataset and predicting the position of molecules of this class in the substructure tree. A molecule from the left-out class is a hit when it is predicted to belong to one of the closest classes in sequence space. The closest classes in sequence space are found using the distance matrix from the multiple sequence alignment. Prediction of the class of a molecule is based on the Euclidean distance in substructure space. This distance is calculated as follows: for each substructure, the square of the difference between the relative frequency in a class and the molecule is calculated. The relative frequency of a substructure in a molecule is either 0 for absence, or 1 for presence of the substructure. The square root of the sum of all squared differences is the Euclidean distance between a molecule and a class. The area under the curve (AUC) of the receiver operating characteristic (ROC) plot served as a quality measure of the predictions for a class.

Instead of repeating the substructure mining for every left-out class, a lookup table of substructure occurrence was used. This table related all generated substructures with all molecules in which they occurred. Substructures that had a frequency just above the support threshold in the left-out class were not considered when analysis was performed for molecules of this class.

## Authors' contributions

EH carried out the sequence alignments, frequent substructure mining, analysis and validation, and drafted the manuscript. JEP participated in design of the study and visualization methods, and implementation of analyses. MWB, JRL, and HWTV assisted in study design, interpretation of results, and drafting the manuscript. MTME was involved in algorithm design and data analysis. YO was involved in acquisition of data in GLIDA. APIJ and AB participated in study design and coordination and helped to draft the manuscript. All authors read and approved the final manuscript.

## Supplementary Material

Additional file 1 **Phylogenetic trees based on 7TM domain and selected residues**. Phylogenetic trees based on 7TM domain and selected residues. Two sequence-based phylogenetic trees for the set of Class A GPCRs used in this study: the phylogenetic tree based on the multiple sequence alignment of the 7TM domain and the phylogenetic tree based on 30 selected residues described in Surgand *et al. *[15]. Subfamilies are color-coded according to ligand type whereby the broad ligand types applied by in Gloriam *et al. *[17] were used. Legend: red - receptor with aminergic ligands; pink - peptide ligands; green - lipid ligands; dark blue - purinergic P2Y ligands; light blue - adenosine ligands; brown - melatonin ligands. Click here for file

Additional file 2 **Plotted scores for the *leave-one-out *validation**. Plotted scores for the *leave-one-out *validation. The complete set of plotted scores of identified ligands per number of closest neighbors (sequences). For each plot, receptors are ordered along the x-axis (labeled "Number of included receptors") in order of increasing distance in sequence space to the receptor under study. The y-axis (labeled "Ligands identified") indicates the cumulative number of retrieved ligands, normalized linearly to the interval [0;1]. The red curve indicates the number of active ligands that are retrieved when including all (closest) receptors that are listed along the x-axis up to that point. More specifically, the number of correctly predicted ligands is plotted against the number of closely related receptors on which the prediction was based. For example, the plot of the muscarinic acetylcholine receptor M_1 _(CHRM1, third row, third plot from the left) displays a steeply rising curve near the origin, indicating that many of its ligands are retrieved using a small number of closest receptors. The blue diagonal illustrates recovery of ligands when performance is equal to random prediction. The relative area under the curve (AUC) of the red curve is stated at the bottom of each plot. An AUC above 0.5 indicates good performance, while poor performance is indicated by an AUC of 0.5 or below. The plots are sorted according to decreasing (relative) AUC. Click here for file

Additional file 3 **List of GPCRs used in this study**. List of GPCRs used in this study. The list of GPCRs used in this study (Class A, excluding singletons). Only receptors that are human, non-olfactory, and not orphan, were used. For each receptor, the respective (sub) family, gene symbol, official IUPHAR name, and number of ligands are provided. Click here for file
