# Supplementary material for: A novel chemogenomics analysis of G protein-coupled receptors (GPCRs) and their ligands: a potential strategy for receptor de-orphanization
Source: BMC Bioinformatics. 2010 Jun 10;11:316. doi: 10.1186/1471-2105-11-316 (PMC2897831; doi:10.1186/1471-2105-11-316)

Phylogenetic tree based on the 7TM domain sequence alignment

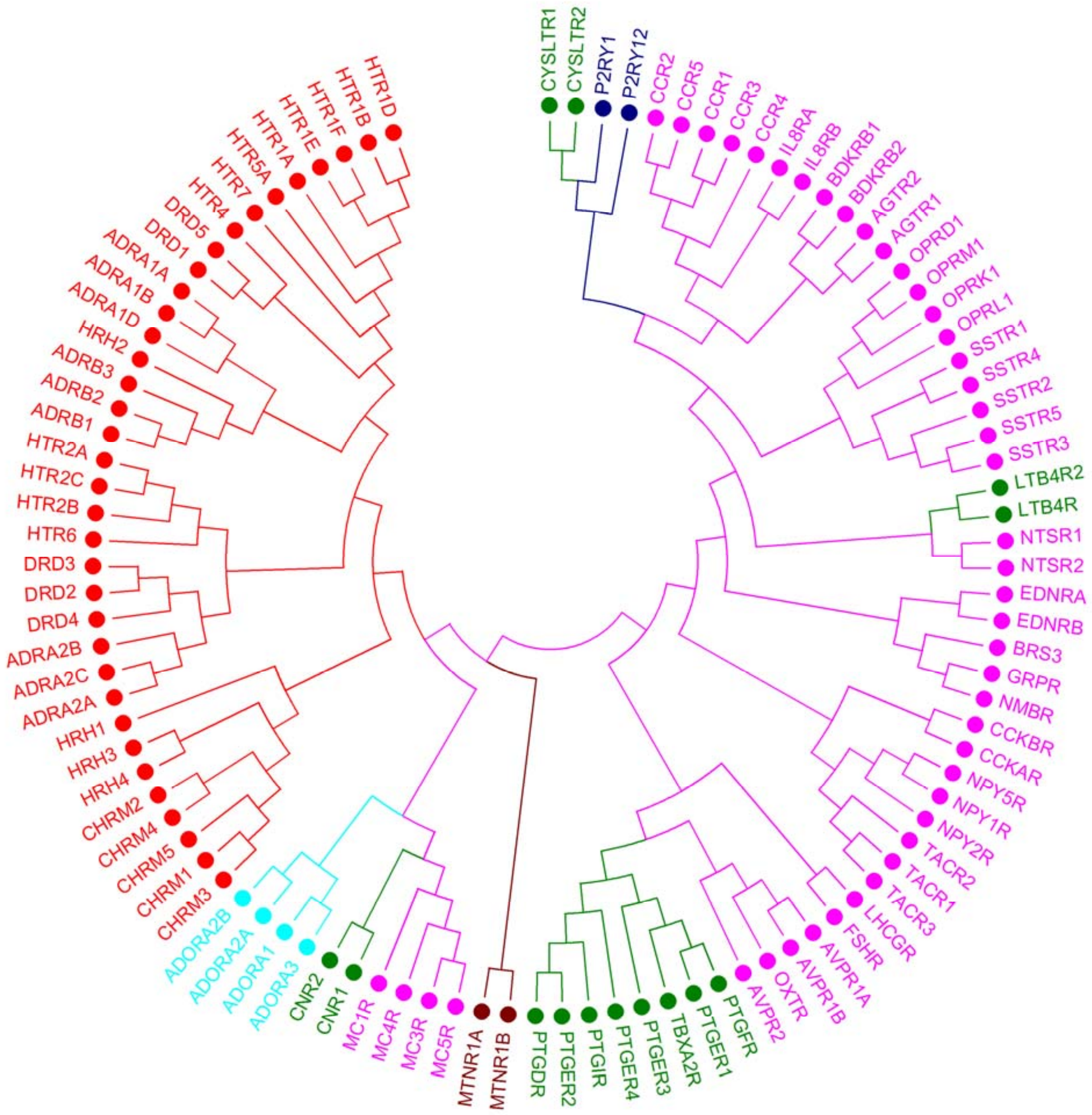

Phylogenetic tree based on 30 selected residues described in Surgand *et al.*

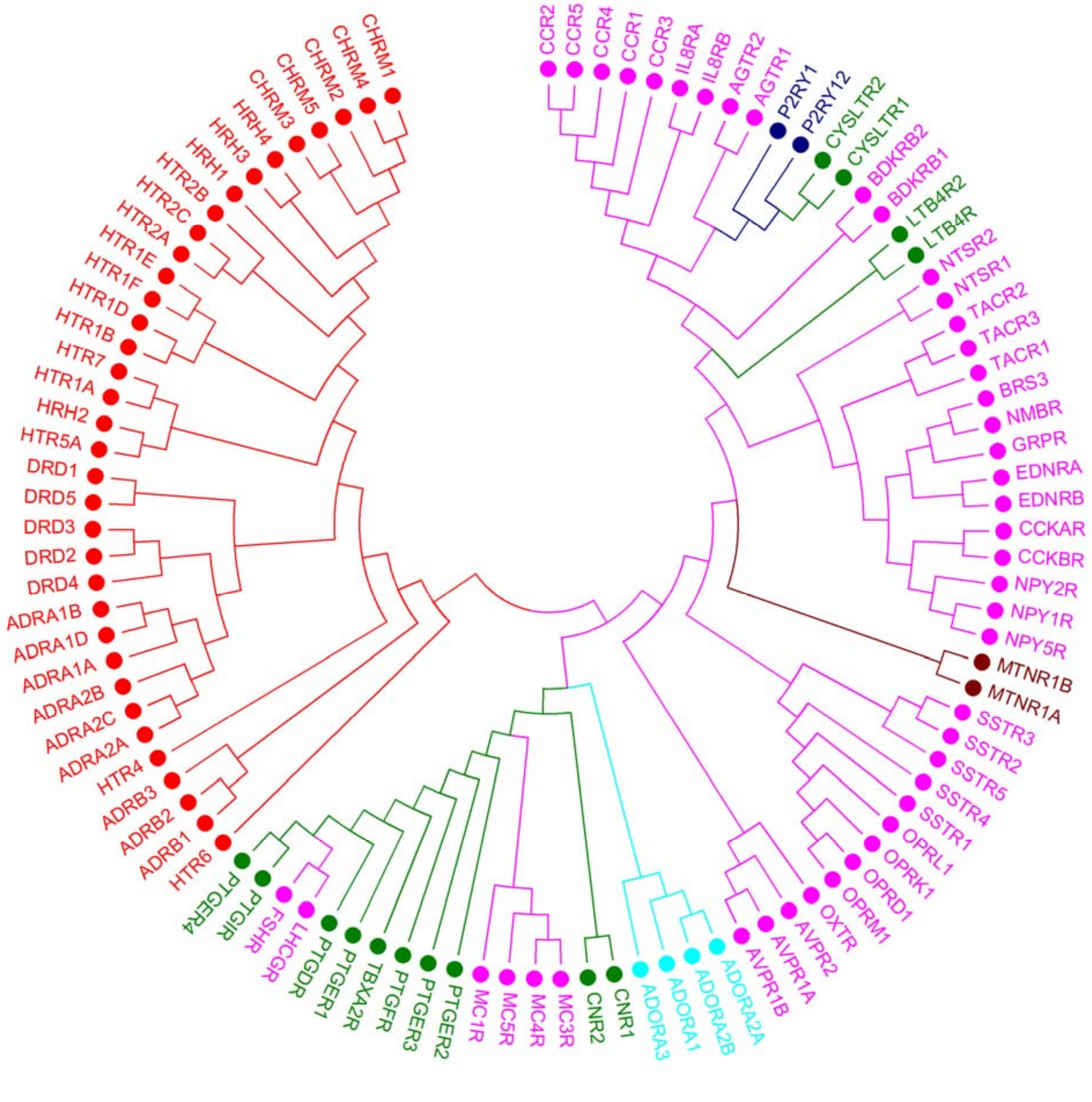

Supplement: Additional file 1 — Phylogenetic trees based on 7TM domain and selected residues. Phylogenetic trees based on 7TM domain and selected residues. Two sequence-based phylogenetic trees for the set of Class A GPCRs used in this study: the phylogenetic tree based on the multiple sequence alignment of the 7TM domain and the phylogenetic tree based on 30 selected residues described in Surgand et al. [15]. Subfamilies are color-coded according to ligand type whereby the broad ligand types applied by in Gloriam et al. [17] were used. Legend: red - receptor with aminergic ligands; pink - peptide ligands; green - lipid ligands; dark blue - purinergic P2Y ligands; light blue - adenosine ligands; brown - melatonin ligands. [file 1471-2105-11-316-S1.PDF]
