# Supplementary material for: A novel chemogenomics analysis of G protein-coupled receptors (GPCRs) and their ligands: a potential strategy for receptor de-orphanization
Source: BMC Bioinformatics. 2010 Jun 10;11:316. doi: 10.1186/1471-2105-11-316 (PMC2897831; doi:10.1186/1471-2105-11-316)
Supplement: Additional file 2 — Plotted scores for the leave-one-out validation. Plotted scores for the leave-one-out validation. The complete set of plotted scores of identified ligands per number of closest neighbors (sequences). For each plot, receptors are ordered along the x-axis (labeled "Number of included receptors") in order of increasing distance in sequence space to the receptor under study. The y-axis (labeled "Ligands identified") indicates the cumulative number of retrieved ligands, normalized linearly to the interval [0;1]. The red curve indicates the number of active ligands that are retrieved when including all (closest) receptors that are listed along the x-axis up to that point. More specifically, the number of correctly predicted ligands is plotted against the number of closely related receptors on which the prediction was based. For example, the plot of the muscarinic acetylcholine receptor M1 (CHRM1, third row, third plot from the left) displays a steeply rising curve near the origin, indicating that many of its ligands are retrieved using a small number of closest receptors. The blue diagonal illustrates recovery of ligands when performance is equal to random prediction. The relative area under the curve (AUC) of the red curve is stated at the bottom of each plot. An AUC above 0.5 indicates good performance, while poor performance is indicated by an AUC of 0.5 or below. The plots are sorted according to decreasing (relative) AUC. [file 1471-2105-11-316-S2.PDF]

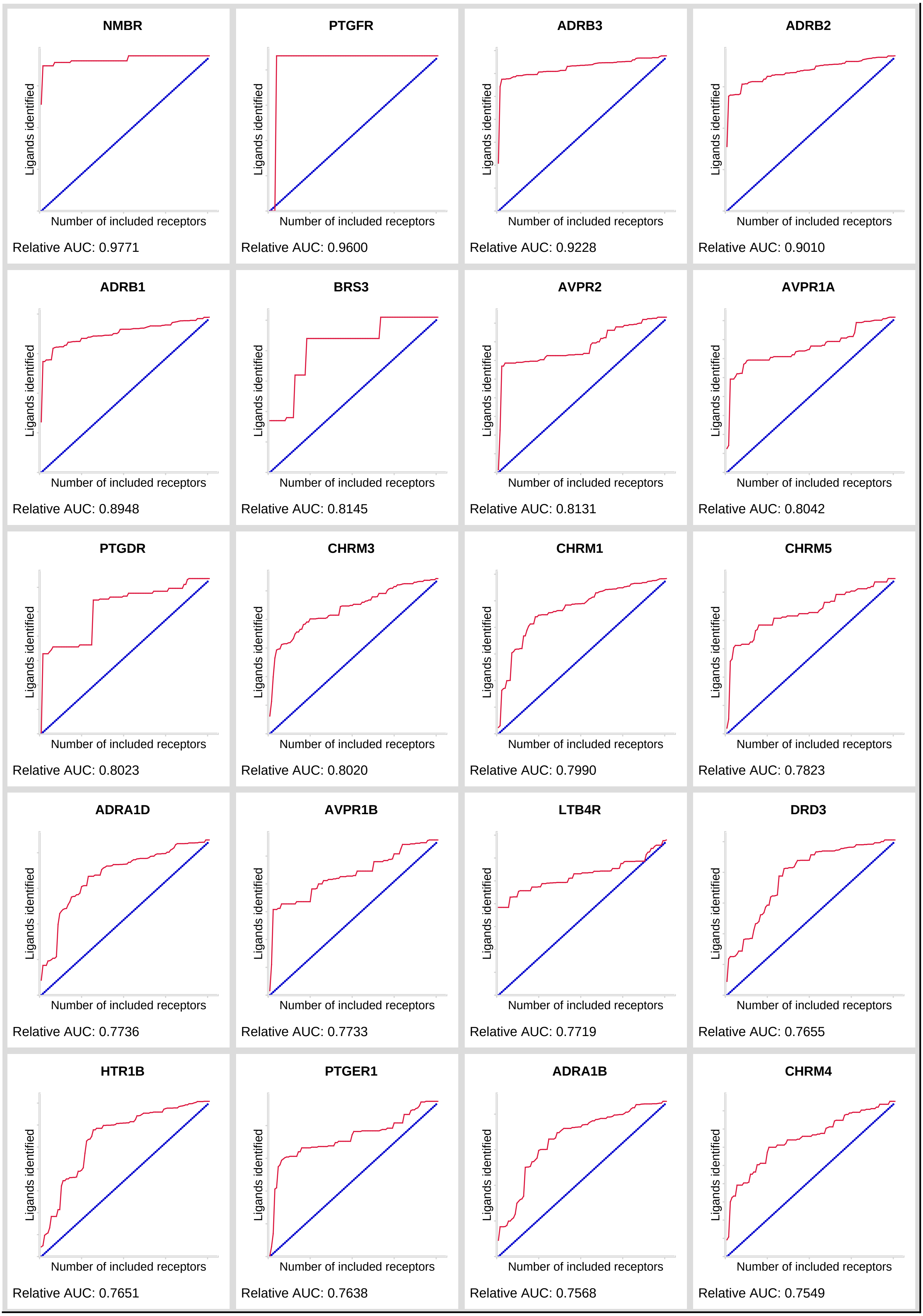
NMBR
Ligands identified
Number of included receptors
Relative AUC: 0.9771
PTGFR
Ligands identified
Number of included receptors
Relative AUC: 0.9600
ADRB3
Ligands identified
Number of included receptors
Relative AUC: 0.9228
ADRB2
Ligands identified
Number of included receptors
Relative AUC: 0.9010
ADRB1
Ligands identified
Number of included receptors
Relative AUC: 0.8948
BRS3
Ligands identified
Number of included receptors
Relative AUC: 0.8145
AVPR2
Ligands identified
Number of included receptors
Relative AUC: 0.8131
AVPR1A
Ligands identified
Number of included receptors
Relative AUC: 0.8042
PTGDR
Ligands identified
Number of included receptors
Relative AUC: 0.8023
CHRM3
Ligands identified
Number of included receptors
Relative AUC: 0.8020
CHRM1
Ligands identified
Number of included receptors
Relative AUC: 0.7990
CHRM5
Ligands identified
Number of included receptors
Relative AUC: 0.7823
ADRA1D
Ligands identified
Number of included receptors
Relative AUC: 0.7736
AVPR1B
Ligands identified
Number of included receptors
Relative AUC: 0.7733
LTB4R
Ligands identified
Number of included receptors
Relative AUC: 0.7719
DRD3
Ligands identified
Number of included receptors
Relative AUC: 0.7655
HTR1B
Ligands identified
Number of included receptors
Relative AUC: 0.7651
PTGER1
Ligands identified
Number of included receptors
Relative AUC: 0.7638
ADRA1B
Ligands identified
Number of included receptors
Relative AUC: 0.7568
CHRM4
Ligands identified
Number of included receptors
Relative AUC: 0.7549

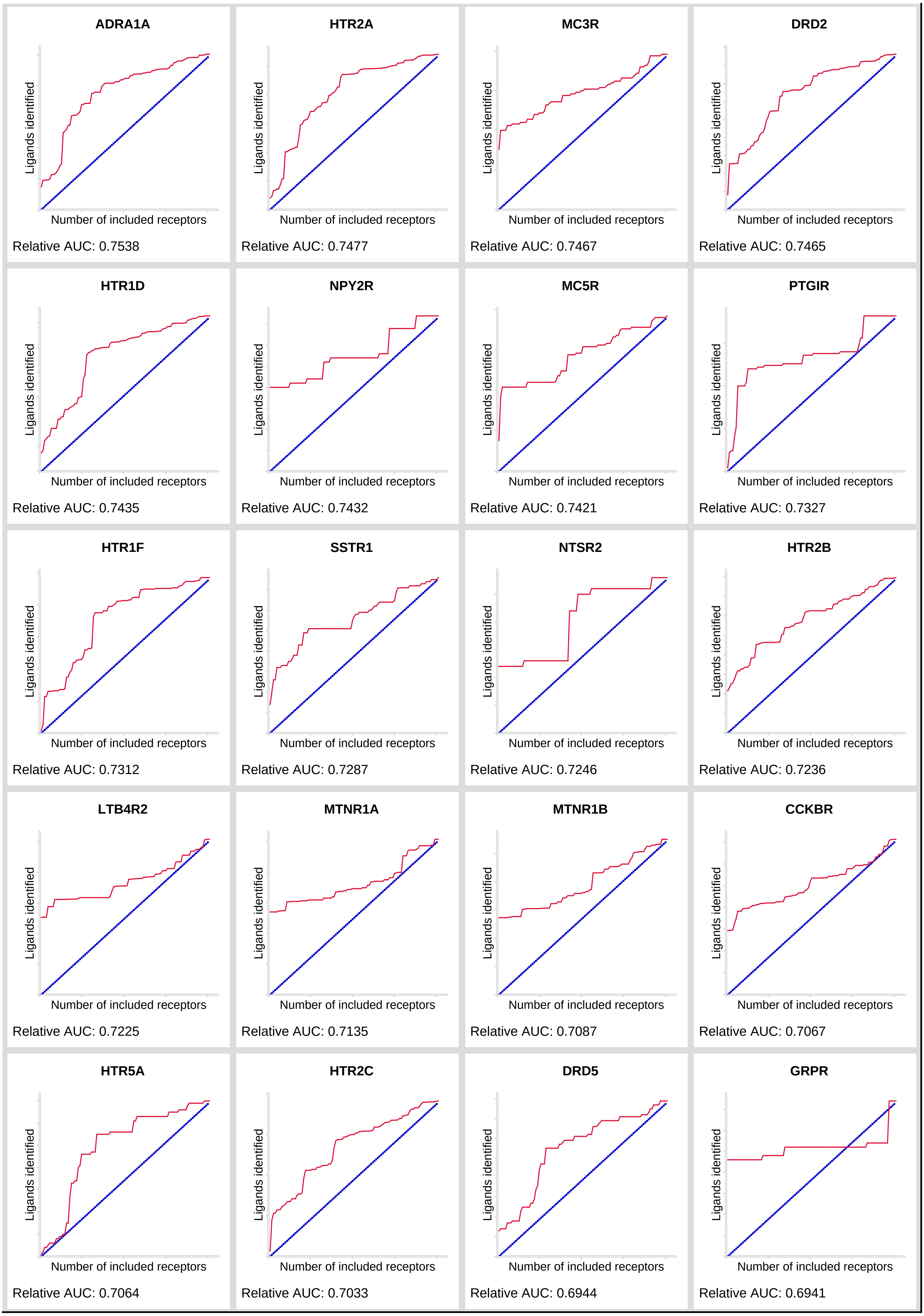
ADRA1A
Ligands identified
Number of included receptors
Relative AUC: 0.7538
HTR2A
Ligands identified
Number of included receptors
Relative AUC: 0.7477
MC3R
Ligands identified
Number of included receptors
Relative AUC: 0.7467
DRD2
Ligands identified
Number of included receptors
Relative AUC: 0.7465
HTR1D
Ligands identified
Number of included receptors
Relative AUC: 0.7435
NPY2R
Ligands identified
Number of included receptors
Relative AUC: 0.7432
MC5R
Ligands identified
Number of included receptors
Relative AUC: 0.7421
PTGIR
Ligands identified
Number of included receptors
Relative AUC: 0.7327
HTR1F
Ligands identified
Number of included receptors
Relative AUC: 0.7312
SSTR1
Ligands identified
Number of included receptors
Relative AUC: 0.7287
NTSR2
Ligands identified
Number of included receptors
Relative AUC: 0.7246
HTR2B
Ligands identified
Number of included receptors
Relative AUC: 0.7236
LTB4R2
Ligands identified
Number of included receptors
Relative AUC: 0.7225
MTNR1A
Ligands identified
Number of included receptors
Relative AUC: 0.7135
MTNR1B
Ligands identified
Number of included receptors
Relative AUC: 0.7087
CCKBR
Ligands identified
Number of included receptors
Relative AUC: 0.7067
HTR5A
Ligands identified
Number of included receptors
Relative AUC: 0.7064
HTR2C
Ligands identified
Number of included receptors
Relative AUC: 0.7033
DRD5
Ligands identified
Number of included receptors
Relative AUC: 0.6944
GRPR
Ligands identified
Number of included receptors
Relative AUC: 0.6941

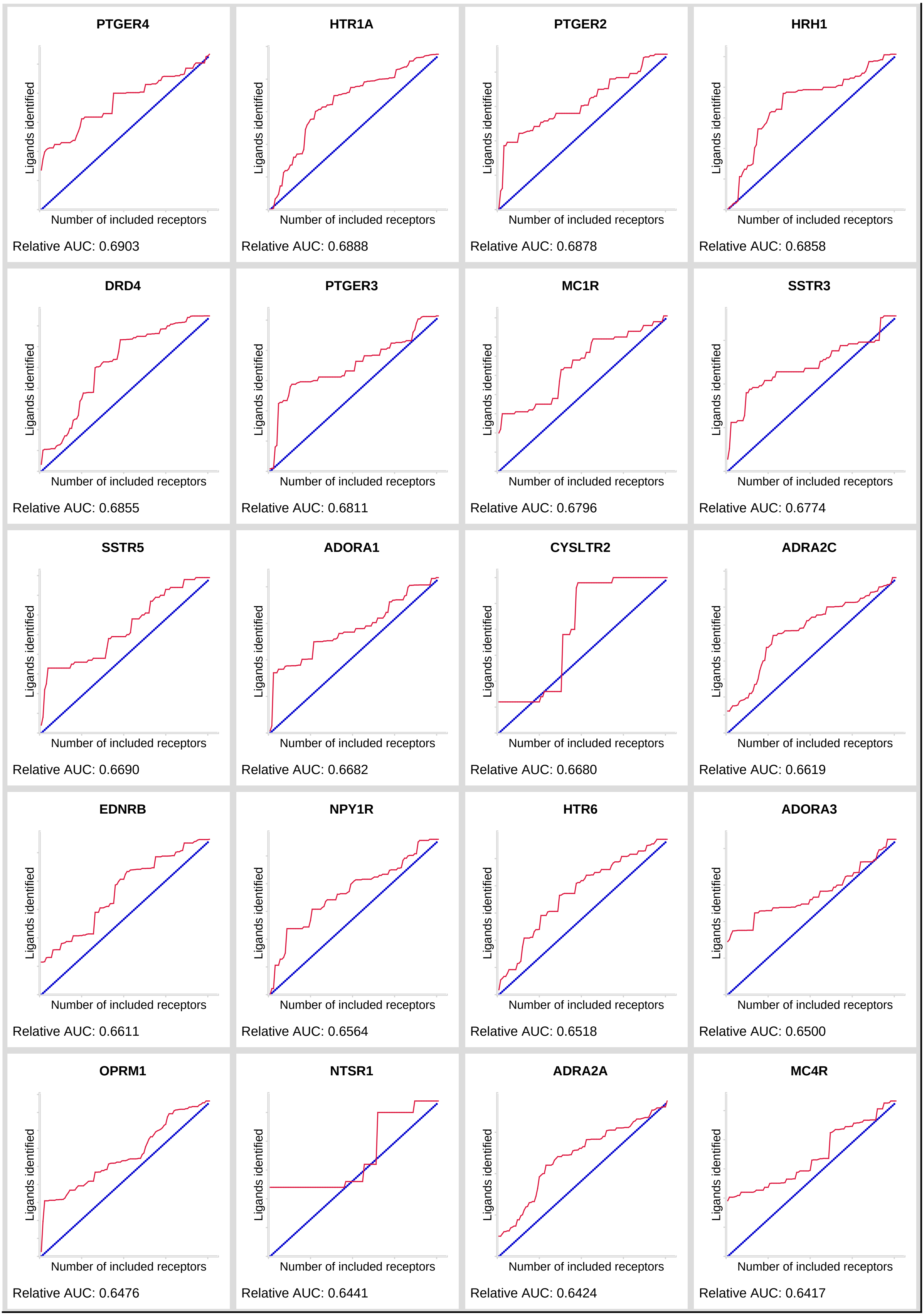
PTGER4
Ligands identified
Number of included receptors
Relative AUC: 0.6903
HTR1A
Ligands identified
Number of included receptors
Relative AUC: 0.6888
PTGER2
Ligands identified
Number of included receptors
Relative AUC: 0.6878
HRH1
Ligands identified
Number of included receptors
Relative AUC: 0.6858
DRD4
Ligands identified
Number of included receptors
Relative AUC: 0.6855
PTGER3
Ligands identified
Number of included receptors
Relative AUC: 0.6811
MC1R
Ligands identified
Number of included receptors
Relative AUC: 0.6796
SSTR3
Ligands identified
Number of included receptors
Relative AUC: 0.6774
SSTR5
Ligands identified
Number of included receptors
Relative AUC: 0.6690
ADORA1
Ligands identified
Number of included receptors
Relative AUC: 0.6682
CYSLTR2
Ligands identified
Number of included receptors
Relative AUC: 0.6680
ADRA2C
Ligands identified
Number of included receptors
Relative AUC: 0.6619
EDNRB
Ligands identified
Number of included receptors
Relative AUC: 0.6611
NPY1R
Ligands identified
Number of included receptors
Relative AUC: 0.6564
HTR6
Ligands identified
Number of included receptors
Relative AUC: 0.6518
ADORA3
Ligands identified
Number of included receptors
Relative AUC: 0.6500
OPRM1
Ligands identified
Number of included receptors
Relative AUC: 0.6476
NTSR1
Ligands identified
Number of included receptors
Relative AUC: 0.6441
ADRA2A
Ligands identified
Number of included receptors
Relative AUC: 0.6424
MC4R
Ligands identified
Number of included receptors
Relative AUC: 0.6417

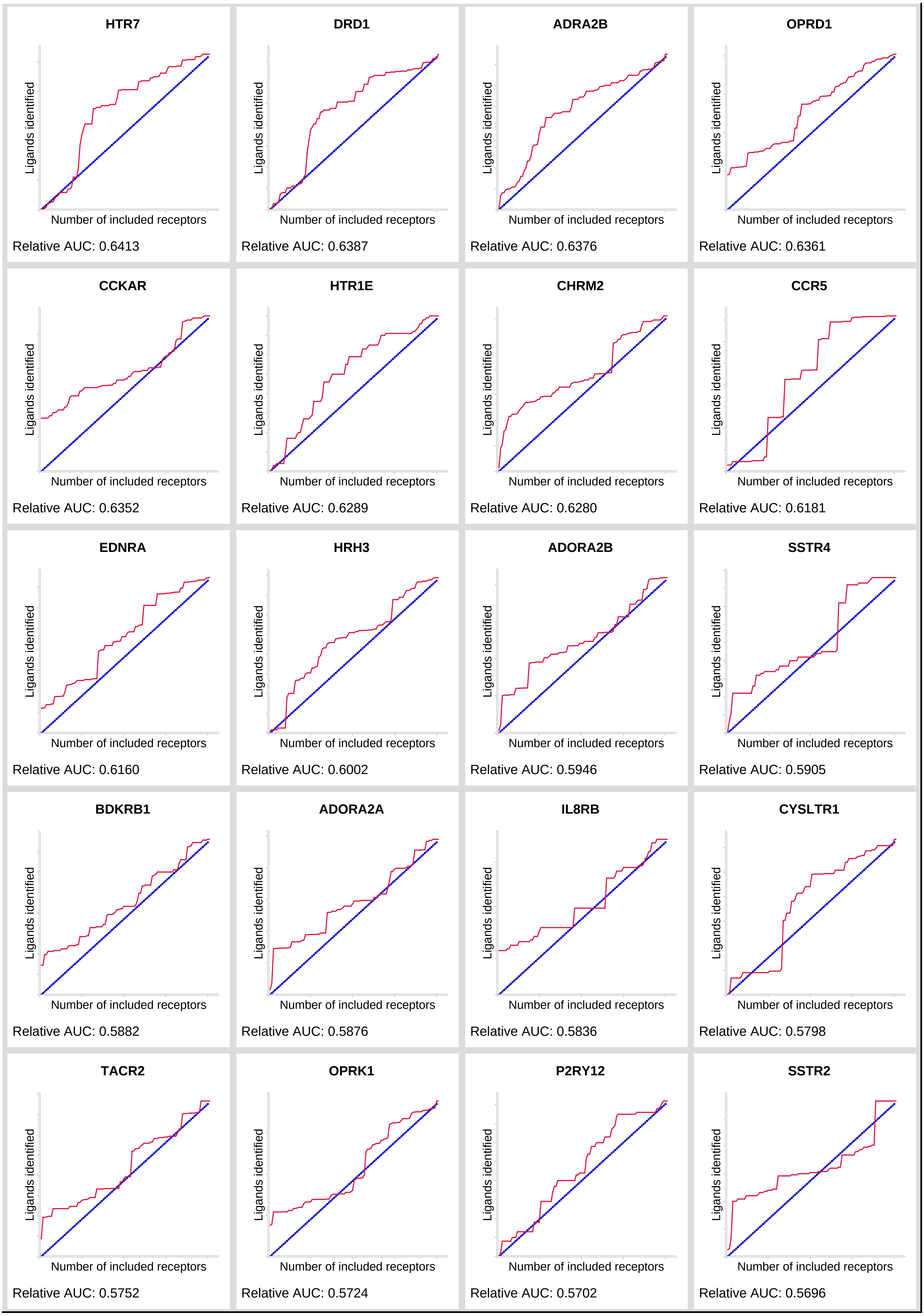
HTR7
Ligands identified
Number of included receptors
Relative AUC: 0.6413
DRD1
Ligands identified
Number of included receptors
Relative AUC: 0.6387
ADRA2B
Ligands identified
Number of included receptors
Relative AUC: 0.6376
OPRD1
Ligands identified
Number of included receptors
Relative AUC: 0.6361
CCKAR
Ligands identified
Number of included receptors
Relative AUC: 0.6352
HTR1E
Ligands identified
Number of included receptors
Relative AUC: 0.6289
CHRM2
Ligands identified
Number of included receptors
Relative AUC: 0.6280
CCR5
Ligands identified
Number of included receptors
Relative AUC: 0.6181
EDNRA
Ligands identified
Number of included receptors
Relative AUC: 0.6160
HRH3
Ligands identified
Number of included receptors
Relative AUC: 0.6002
ADORA2B
Ligands identified
Number of included receptors
Relative AUC: 0.5946
SSTR4
Ligands identified
Number of included receptors
Relative AUC: 0.5905
BDKRB1
Ligands identified
Number of included receptors
Relative AUC: 0.5882
ADORA2A
Ligands identified
Number of included receptors
Relative AUC: 0.5876
IL8RB
Ligands identified
Number of included receptors
Relative AUC: 0.5836
CYSLTR1
Ligands identified
Number of included receptors
Relative AUC: 0.5798
TACR2
Ligands identified
Number of included receptors
Relative AUC: 0.5752
OPRK1
Ligands identified
Number of included receptors
Relative AUC: 0.5724
P2RY12
Ligands identified
Number of included receptors
Relative AUC: 0.5702
SSTR2
Ligands identified
Number of included receptors
Relative AUC: 0.5696

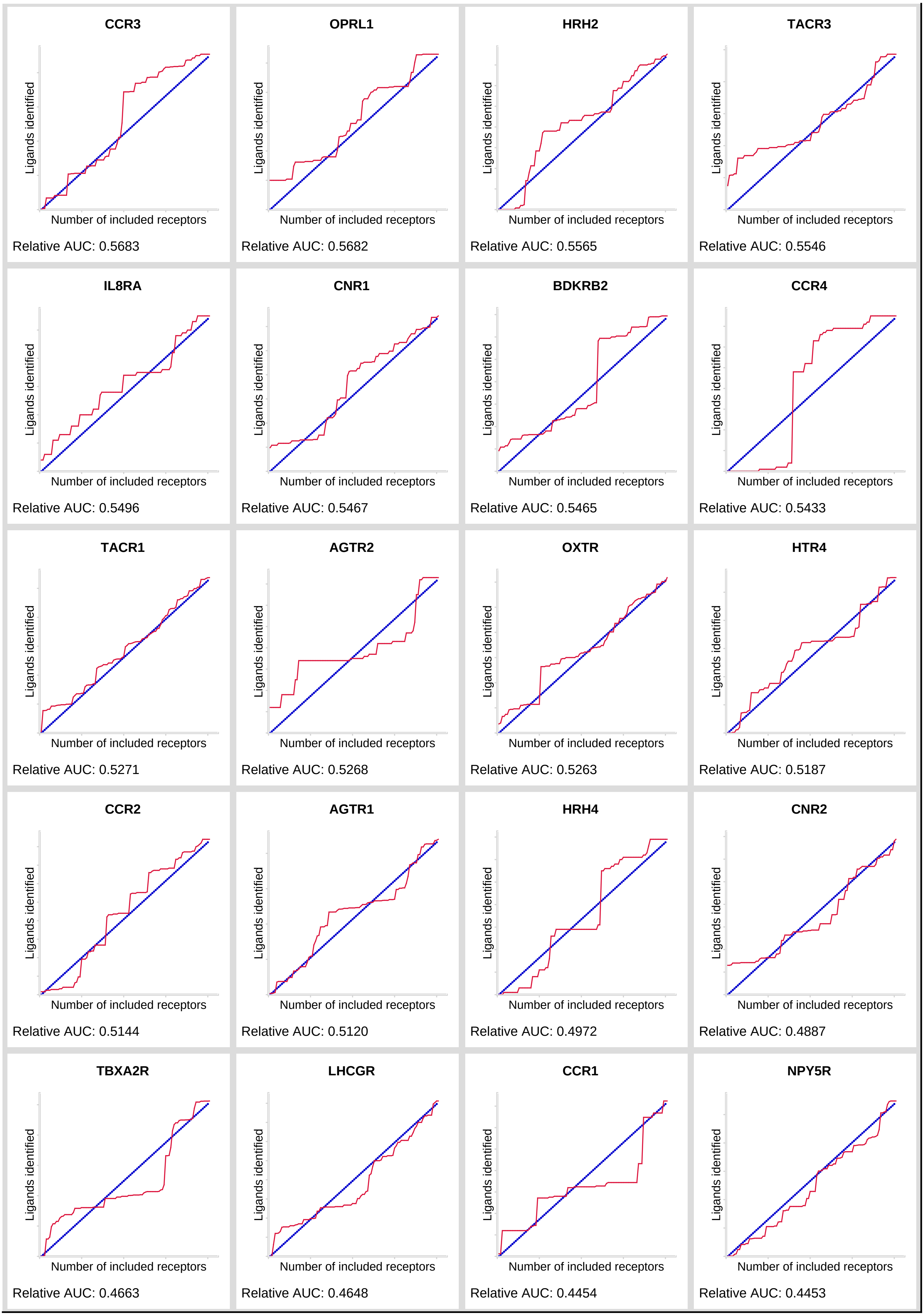
CCR3
Ligands identified
Number of included receptors
Relative AUC: 0.5683
OPRL1
Ligands identified
Number of included receptors
Relative AUC: 0.5682
HRH2
Ligands identified
Number of included receptors
Relative AUC: 0.5565
TACR3
Ligands identified
Number of included receptors
Relative AUC: 0.5546
IL8RA
Ligands identified
Number of included receptors
Relative AUC: 0.5496
CNR1
Ligands identified
Number of included receptors
Relative AUC: 0.5467
BDKRB2
Ligands identified
Number of included receptors
Relative AUC: 0.5465
CCR4
Ligands identified
Number of included receptors
Relative AUC: 0.5433
TACR1
Ligands identified
Number of included receptors
Relative AUC: 0.5271
AGTR2
Ligands identified
Number of included receptors
Relative AUC: 0.5268
OXTR
Ligands identified
Number of included receptors
Relative AUC: 0.5263
HTR4
Ligands identified
Number of included receptors
Relative AUC: 0.5187
CCR2
Ligands identified
Number of included receptors
Relative AUC: 0.5144
AGTR1
Ligands identified
Number of included receptors
Relative AUC: 0.5120
HRH4
Ligands identified
Number of included receptors
Relative AUC: 0.4972
CNR2
Ligands identified
Number of included receptors
Relative AUC: 0.4887
TBXA2R
Ligands identified
Number of included receptors
Relative AUC: 0.4663
LHCGR
Ligands identified
Number of included receptors
Relative AUC: 0.4648
CCR1
Ligands identified
Number of included receptors
Relative AUC: 0.4454
NPY5R
Ligands identified
Number of included receptors
Relative AUC: 0.4453

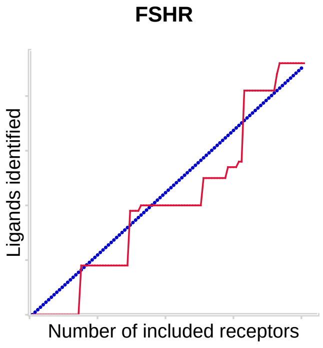

Relative AUC: 0.4428

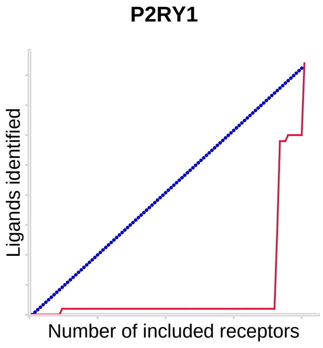

Relative AUC: 0.0857
