# Supplementary material for: A novel chemogenomics analysis of G protein-coupled receptors (GPCRs) and their ligands: a potential strategy for receptor de-orphanization
Source: BMC Bioinformatics. 2010 Jun 10;11:316. doi: 10.1186/1471-2105-11-316 (PMC2897831; doi:10.1186/1471-2105-11-316)
Supplement: Additional file 3 — List of GPCRs used in this study. List of GPCRs used in this study. The list of GPCRs used in this study (Class A, excluding singletons). Only receptors that are human, non-olfactory, and not orphan, were used. For each receptor, the respective (sub) family, gene symbol, official IUPHAR name, and number of ligands are provided. [file 1471-2105-11-316-S3.PDF]

Table 1 - List of GPCRs used in this study (Class A, excluding singletons).

Only receptors that were human, non-olfactory, and not orphan, were used. For each receptor, the respective (sub) family, gene symbol, official IUPHAR name, and number of ligands are provided.

Family	Gene symbol	Official IUPHAR name	Number of ligands
5-Hydroxytryptamine receptors	HTR1A	5-HT_{1A}	2382
5-Hydroxytryptamine receptors	HTR1B	5-HT_{1B}	708
5-Hydroxytryptamine receptors	HTR1D	5-HT_{1D}	1568
5-Hydroxytryptamine receptors	HTR1E	5-HT_{1E}	80
5-Hydroxytryptamine receptors	HTR1F	5-HT_{1F}	243
5-Hydroxytryptamine receptors	HTR2A	5-HT_{2A}	1353
5-Hydroxytryptamine receptors	HTR2B	5-HT_{2B}	398
5-Hydroxytryptamine receptors	HTR2C	5-HT_{2C}	958
5-Hydroxytryptamine receptors	HTR4	5-HT_4	552
5-Hydroxytryptamine receptors	HTR5A	5-HT_{5A}	70
5-Hydroxytryptamine receptors	HTR6	5-HT_6	571
5-Hydroxytryptamine receptors	HTR7	5-HT_7	263
Acetylcholine receptors (muscarinic)	CHRM1	M_1	1459
Acetylcholine receptors (muscarinic)	CHRM2	M_2	611
Acetylcholine receptors (muscarinic)	CHRM3	M_3	543
Acetylcholine receptors (muscarinic)	CHRM4	M_4	213

Acetylcholine receptors (muscarinic)	CHRM5	M_5	137
Adenosine receptors	ADORA1	A_1	1063
Adenosine receptors	ADORA2A	A_{2A}	1223
Adenosine receptors	ADORA2B	A_{2B}	1050
Adenosine receptors	ADORA3	A_3	1222
Adrenoceptors	ADRA1A	α_{1A}	1256
Adrenoceptors	ADRA1B	α_{1B}	1090
Adrenoceptors	ADRA1D	α_{1D}	1090
Adrenoceptors	ADRA2A	α_{2A}	939
Adrenoceptors	ADRA2B	α_{2B}	751
Adrenoceptors	ADRA2C	α_{2C}	864
Adrenoceptors	ADRB1	β_1	980
Adrenoceptors	ADRB2	β_2	937
Adrenoceptors	ADRB3	β_3	1693
Angiotensin receptors	AGTR1	AT_1	2199
Angiotensin receptors	AGTR2	AT_2	73
Bombesin receptors	BRS3	BB_3	51
Bombesin receptors	GRPR	BB_2	37
Bombesin receptors	NMBR	BB_1	93
Bradykinin receptors	BDKRB1	B_1	190
Bradykinin receptors	BDKRB2	B_2	347
Cannabinoid receptors	CNR1	CB_1	322
Cannabinoid receptors	CNR2	CB_2	344

Chemokine receptors	CCR1	CCR1	181
Chemokine receptors	CCR2	CCR2	210
Chemokine receptors	CCR3	CCR3	454
Chemokine receptors	CCR4	CCR4	75
Chemokine receptors	CCR5	CCR5	719
Chemokine receptors	IL8RA	CXCR1	55
Chemokine receptors	IL8RB	CXCR2	88
Cholecystokinin receptors	CCKAR	CCK_1	453
Cholecystokinin receptors	CCKBR	CCK_2	713
Dopamine receptors	DRD1	D_1	367
Dopamine receptors	DRD2	D_2	2159
Dopamine receptors	DRD3	D_3	1264
Dopamine receptors	DRD4	D_4	1870
Dopamine receptors	DRD5	D_5	79
Endothelin receptors	EDNRA	ET_A	1863
Endothelin receptors	EDNRB	ET_B	1368
Glycoprotein hormone receptors	FSHR	FSH	46
Glycoprotein hormone receptors	LHCGR	LH	406
Histamine receptors	HRH1	H_1	254
Histamine receptors	HRH2	H_2	188
Histamine receptors	HRH3	H_3	789
Histamine receptors	HRH4	H_4	69
Leukotriene receptors	CYSLTR1	$CysLT_1$	634

Leukotriene receptors	CYSLTR2	$CysLT_2$	30
Leukotriene receptors	LTB4R	BLT_1	679
Leukotriene receptors	LTB4R2	BLT_2	509
Melanocortin receptors	MC1R	MC_1	81
Melanocortin receptors	MC3R	MC_3	98
Melanocortin receptors	MC4R	MC_4	533
Melanocortin receptors	MC5R	MC_5	96
Melatonin receptors	MTNR1A	MT_1	507
Melatonin receptors	MTNR1B	MT_2	551
Neuropeptide Y receptors	NPY1R	Y_1	280
Neuropeptide Y receptors	NPY2R	Y_2	37
Neuropeptide Y receptors	NPY5R	Y_5	548
Neurotensin receptors	NTSR1	NTS_1	27
Neurotensin receptors	NTSR2	NTS_2	28
Opioid receptors	OPRD1	δ	1011
Opioid receptors	OPRK1	κ	945
Opioid receptors	OPRL1	NOP	265
Opioid receptors	OPRM1	μ	864
P2Y receptors	P2RY1	$P2Y_1$	42
P2Y receptors	P2RY12	$P2Y_{12}$	82
Prostanoid receptors	PTGDR	DP_1	159
Prostanoid receptors	PTGER1	EP_1	237
Prostanoid receptors	PTGER2	EP_2	226

Prostanoid receptors	PTGER3	EP_3	257
Prostanoid receptors	PTGER4	EP_4	267
Prostanoid receptors	PTGFR	FP	44
Prostanoid receptors	PTGIR	IP_1	91
Prostanoid receptors	TBXA2R	TP	1281
Somatostatin receptors	SSTR1	sst_1	76
Somatostatin receptors	SSTR2	sst_2	141
Somatostatin receptors	SSTR3	sst_3	89
Somatostatin receptors	SSTR4	sst_4	86
Somatostatin receptors	SSTR5	sst_5	79
Tachykinin receptors	TACR1	NK_1	2686
Tachykinin receptors	TACR2	NK_2	768
Tachykinin receptors	TACR3	NK_3	486
Vasopressin and oxytocin receptors	AVPR1A	V_{1A}	409
Vasopressin and oxytocin receptors	AVPR1B	V_{1B}	279
Vasopressin and oxytocin receptors	AVPR2	V_2	416
Vasopressin and oxytocin receptors	OXTR	OT	309
